# Effects of SARS-CoV-2 Vaccination on Menstrual Cycle: An Italian Survey-Based Study

**DOI:** 10.3390/jcm12247699

**Published:** 2023-12-15

**Authors:** Roberta Granese, Giosuè Giordano Incognito, Ferdinando Antonio Gulino, Giorgia Casiraro, Paola Porcaro, Angela Alibrandi, Canio Martinelli, Alfredo Ercoli

**Affiliations:** 1 Department of Biomedical and Dental Sciences and Morphofunctional Imaging, “G. Martino” University Hospital, 98100 Messina, Italy; 2 Department of General Surgery and Medical Surgical Specialties, University of Catania, 95125 Catania, Italy; giordanoincognito@gmail.com; 3 Unit of Gynecology and Obstetrics, Department of Human Pathology of Adults and Developmental Age, “G. Martino” University Hospital, 98100 Messina, Italy; docferdi@hotmail.it (F.A.G.); giorgiacasiraro@gmail.com (G.C.); caniomartinelli.md@gmail.com (C.M.); aercoli@unime.it (A.E.); 4 Department of Obstetrics and Gynecology, “Santa Maria Ungheretti” Hospital, 89024 Polistena, Italy; paolaporcaro1210@gmail.com; 5 Unit of Statistical and Mathematical Sciences, Department of Economics, University of Messina, 98100 Messina, Italy; aalibrandi@unime.it

**Keywords:** SARS-CoV-2, COVID-19, vaccination, menstrual cycle

## Abstract

Vaccination against SARS-CoV-2 has played a critical role in controlling the spread of the pandemic. The main side effects of SARS-CoV-2 vaccination include fever and fatigue; however, the potential impacts on menstrual cycles are to be determined. Given the limited number of studies suggesting menstrual changes post vaccination, this study investigates the correlation between COVID-19 vaccines and menstrual cycle changes in fertile-aged Italian women. A questionnaire was distributed from 1 October to 31 November 2022, focusing on menstrual rhythm and flow changes post vaccination. The analysis involved 471 participants. The study observed a shift from a regular to an irregular menstrual rhythm (*p* < 0.001), and changes in menstrual duration (*p* = 0.008 and *p* < 0.001 for first and second doses, respectively) and flow volume (*p* < 0.001). Most patients with irregular rhythms were vaccinated in the proliferative phase of their cycle. Within six months post vaccination, 74.2% of women with irregular post-vaccination rhythms reported a return to normality. These findings indicate primarily transient menstrual changes following mRNA COVID-19 vaccination, suggesting the vaccines’ safety for women of reproductive age.

## 1. Introduction

Coronavirus Disease 2019 (COVID-19) is an illness caused by severe acute respiratory syndrome coronavirus 2 (SARS-CoV-2), first identified in December 2019. COVID-19 has since become a global pandemic, affecting millions of people worldwide and leading to significant morbidity and mortality. Italy was among the first countries to be heavily impacted by the COVID-19 pandemic, following the initial cases that emerged in Wuhan, China, towards the end of 2019 [1,2].

From a pathogenetic point of view, the virus primarily targets cells expressing angiotensin-converting enzyme 2 (ACE2) receptors, which are abundantly found in the human respiratory epithelium, among other tissues. Utilizing its spike (S) protein, SARS-CoV-2 binds to ACE2 receptors. This is followed by endocytosis, wherein the virus is internalized into the host cell. Once inside the host cell, the viral RNA is released, leveraging the host cell machinery to replicate and transcribe its RNA, consequently synthesizing viral proteins and assembling new virions. As the infection progresses, the host’s immune response is triggered, aiming to eradicate the virus and, in some instances, can become dysregulated, giving rise to a cytokine storm [3]. This heightened immune response can inadvertently cause considerable damage to host tissues, leading to severe symptoms and, in the worst cases, multi-organ failure and death [2].

In response to the unprecedented health crisis, a diverse range of COVID-19 vaccines were developed at an extraordinary pace. Additionally, social isolation measures aimed at mitigating the spread of the virus led to psychological distress, social stress, changes in work and/or study activities, increased sedentary behavior, and, consequently, an uptick in cases of overweight and obesity [4,5].

The SARS-CoV-2 vaccines can be broadly categorized into several types based on their mechanisms of action. The mRNA vaccines, such as those produced by Pfizer-BioNTech and Moderna, represent a groundbreaking approach utilizing messenger RNA to instruct cells to produce the SARS-CoV-2 spike protein, thereby eliciting an immune response [6]. Viral vector vaccines, including those produced by AstraZeneca and Johnson & Johnson, use a modified virus (which is different from the coronavirus) to deliver SARS-CoV-2 genetic material to cells, triggering immunity [7]. Inactivated virus vaccines, like Sinovac’s CoronaVac, contain a killed version of the virus that is unable to cause the disease but is capable of stimulating an immune response [8].

As the global vaccination effort intensified, monitoring and understanding the broader implications of these vaccines became crucial, especially in terms of potential side effects. According to the Italian Medicines Agency (AIFA), the most reported side effects of the vaccines include arm pain, fever, fatigue, and muscle aches. Interestingly, menstrual cycle alterations have not been officially listed as side effects; however, some cases in vaccinated women were reported. This was seen irrespective of the type of vaccine they received, but particularly with mRNA vaccines [9,10,11]. Though these observations have not been widely acknowledged as a standard vaccine side effect, they have sparked important discussions about menstrual health and vaccine safety [12,13,14,15].

Therefore, this study aims to investigate any potential changes in the menstrual cycle after receiving an mRNA vaccine, exploring a relatively under-researched aspect of these vaccine’s effects.

## 2. Materials and Methods

The study was carried out using an online questionnaire titled “Menstrual Cycle Changes and COVID-19 Vaccination: Possible Link or Overestimated?” (Supplementary File S1). This questionnaire, comprising 27 multiple-choice questions, was disseminated online in Italian through social media platforms from 1 October 2022 to 31 November 2022. The study population included all fertile-aged women from various regions of Italy, aged between 18 and 47 years, who had received both the first and the second doses of an mRNA SARS-CoV-2 vaccine. Exclusion criteria were as follows: women below 18 or above 47 years of age; women undergoing any form of hormonal treatment; women who already had irregular menstrual cycles before the pandemic; and pregnant women. The questionnaire was intended for self-administration, and each respondent was allowed to complete it once. The gathered data were anonymized, and the study was conducted in accordance with the Declaration of Helsinki and was approved by the local Ethics Committee. All included women provided their informed consent for data collection and analysis for research purposes before initiating the study.

Multiple sociodemographic variables were scrutinized, such as age, income, education, and changes in work and/or study activity; health-related variables such as weight, physical activity, and stress; reproductive history (pregnancies and abortions); menstrual cycle rhythm; and the characteristics of menstrual flow (duration and quantity) during the pandemic and after the first and second vaccine doses. The cycle rhythm was considered “normal” when it ranged from 25 to 35 days. The duration of the cycle was deemed “normal” when it fell within 4 to 7 days. The amount of menstrual flow was subjectively defined by the patient.

As for patients with irregular post-vaccination rhythms, the menstrual phase in which they were during the vaccine doses was examined. Lastly, it was investigated whether women with irregular post-vaccination rhythms experienced a return to normality within six months after receiving the vaccination.

Categorical and ordinal data were expressed as absolute frequencies and percentages. In order to identify possible significant differences between the two initial timepoints (pre-pandemic versus pre-vaccination), the McNemar test was applied concerning rhythm, which was expressed as dichotomous variables (regular or irregular), and the Wilcoxon signed-rank test was applied concerning ordinal data such as duration and menstrual flow volume. So, since the two timepoints were found to be similar, the statistical analysis was performed examining three timepoints: A. during the pre-vaccination pandemic phase; B. after the first vaccination; C. after the second vaccination. More specifically, for the rhythm, the percentage of transition from regularity to irregularity was calculated and compared at two timepoints by using a comparison of proportion test. For length, the proportion of transition from 4–7 days to 1–3 days and, in addition, from 4–7 days to >7 days was calculated and compared at two timepoints, by using a comparison of proportion test. Finally, for the quantity, the proportion of transition from normal to light and, also, from normal to heavy was calculated and compared at two timepoints, by using the comparison of proportion test. For these analyses of comparison, Bonferroni’s correction was used, so the adjusted alpha level for multiplicity control was obtained by dividing the significance alpha level for the number of possible two-by-two comparisons between timepoints (0.050/3 = 0.017). Statistical analyses were performed using IBM SPSS for Windows, Version 22 (IBM Corp., Armonk, NY, USA). A *p*-value lower than 0.05 was considered to be statistically significant.

## 3. Results

A total of 755 women responded to the questionnaire. Upon applying the inclusion criteria, 471 patients who had regular menstrual cycles and received both the first and second doses of the SARS-CoV-2 vaccine were included in the study, and their questionnaire responses were analyzed.

The average age of the participants was 32 ± 7.4 years. Only 13% had contracted a SARS-CoV-2 infection at the time of completing the questionnaire. Regarding family income, excluding students (21%), this factor remained unchanged for 48.7%, increased for 12.5%, and decreased for 16.1% of the participants; 1.7% reported job loss due to the pandemic. About 65.7% of the women held a university degree, 31.3% had a high school diploma, and the remaining 3% had only completed middle school. During the pandemic, work and/or study activities increased for 45.2%, remained the same for 29%, and decreased for 16.3% of the participants. Furthermore, 9.5% reported not working. Weight increased for 37.5%, remained the same for 42.3%, and decreased for 20.2% of the women. In terms of physical activity, 46% reported a decrease, 33% had no change, and 21% reported an increase. Stress levels rose for 79%, decreased for 6.2%, and remained unchanged for 14.8% of respondents. About 24.9% of the women had experienced pregnancy, while 75.1% reported no prior pregnancies. Additionally, 9.9% reported having had at least one abortion before the pandemic.

A comparison concerning the menstrual cycle rhythm and the menstrual flow duration and quantity during the pandemic before vaccination, after the first dose, and after the second dose of vaccination is presented in Table 1, Table 2 and Table 3, respectively.

No statistically significant differences were found between the pre-pandemic and pre-vaccination phases regarding menstrual cycle rhythm, duration, and quantity (*p* = 0.108, *p* = 0.119, and 0.096, respectively) (Table 4), excluding the direct effect of the pandemic.

Therefore, since the pre-pandemic and pre-vaccination phases were found to be similar, the statistical analysis was performed examining three timepoints (Table 5).

The results revealed a significant shift from a regular to an irregular rhythm between the pre-vaccination phase and after both the first and second doses (both *p* < 0.001). However, no differences were noted between the post-first-dose and post-second-dose phases (*p* = 0.139).

A significant change in duration, from regular to 1–3 days of menstrual flow, was observed between the pre-vaccination phase and after both the first (*p* = 0.008) and second doses (*p* < 0.001). No difference was detected between the post-first-dose and the post-second-dose phases (*p* = 0.242). Similarly, there was a notable change from regular to >7 days between the pre-vaccination phase and after the first (*p* < 0.024) and second doses (*p* < 0.001). Yet again, no difference was observed between the first and second doses (*p* = 0.129).

In terms of menstrual flow volume, a significant change from normal to light was noted between the pre-vaccination phase and after both the first (*p* = 0.001) and second doses (*p* < 0.001). No differences were detected between the two doses (*p* = 0.095). Similarly, the results did not show any significant changes from normal to heavy flow between the pre-vaccination phase and after both doses (both *p* < 0.001), with no observable differences between the two doses (*p* = 0.761).

A total of 74.2% of women with irregular post-vaccination rhythms reported a return to normality within six months after receiving the vaccination.

As for patients with irregular post-vaccination rhythms, the menstrual phase in which they were during the vaccine doses was examined.

Table 6 and Table 7 show the exact menstrual phase in which patients with an irregular rhythm after the first vaccination (*n* = 111) underwent the first vaccination and the exact menstrual phase in which patients with an irregular rhythm after the second vaccination (*n* = 124) underwent the second vaccination. Most of these patients received the vaccine before the 14th day of their cycle; this applies to both the first and second doses (73.9% and 75.8%, respectively).

## 4. Discussion

The menstrual cycle is regulated by a complex interaction of hormones that relate to various systems, including the nervous, immune, vascular, and coagulation systems, which in turn depend on the hypothalamus–pituitary–ovary–endometrium axis [16].

These results did not highlight any significant differences between the pre-pandemic and pre-vaccination phases regarding menstrual cycle rhythm, duration, and menstrual flow volume, excluding the direct effect of the pandemic. On the contrary, it was found that there was a significant change in cycle rhythm and characteristics of menstrual flow in terms of duration and quantity between the pre-vaccination and post vaccination phases. 

However, most women with an irregular cycle rhythm reported a return to normality within six months post vaccination. Additionally, we examined the menstrual phase the patients were in during the first and second vaccine doses. Most of them received the both the first and second dose of the vaccine in the proliferative phase of their cycle. We hypothesized that vaccination induced a lack of ovulation and, therefore, a subsequent irregularity of the cycle for the following month or months.

Menstrual changes after vaccination do not appear to be uncommon, as these alterations have also been observed after vaccination for other viruses, such as Human papillomavirus (HPV) [17,18]. From a pathogenic standpoint, such disturbances could likely be attributed to an inflammatory/immunological reaction stemming from the adjuvants in vaccines [19]. However, the SARS-CoV-2 spike glycoprotein could also play a pathogenic role, as similar changes in menstrual cycles have been recorded in some cases of COVID-19 [20]. Moreover, the spread of the spike protein in women’s tissues, released after mRNA vaccination, might also interfere with the endocrine homeostasis of the menstrual cycle, since the use of combined oral contraceptives has been associated with a lower likelihood of reporting menstrual changes [10]. These findings further confirm the protective effect of estrogens in mitigating the severity of clinical outcomes related to COVID-19 [21].

Furthermore, strategies for controlling COVID-19 infection, such as social distancing, have led to an increase in psychological stress [5]. Some studies have also reported a correlation between the pandemic, physical activity, and weight gain [22], as also confirmed by this study. The body responds to stress by increasing the production of GH, ACTH, and prolactin, and by activating the sympathetic system. Specifically, the production of glucocorticoids and catecholamines is the pivotal moment through which metabolic adaptive responses are realized. The presence of psychological alterations, recognized as stressors, activates the hypothalamus–pituitary–adrenal axis. In the presence of an acute stimulus that resolves, there is a simple increase in cortisol secretion. However, if the stressor stimulus persists, there is a more moderate activation and cortisol secretion is slightly elevated without loss of the circadian rhythm, which is instead altered by chronic stressors of adequate intensity, compromising reproductive function as well. Psychological stress is a known risk factor for hypothalamic anovulation, in which there may be a functional alteration in the secretion of GnRH, resulting in a disturbance of menstrual cyclicity [23]. The absence of ovulation due to functional hypothalamic origin is the result of psychoneuroendocrine integration, where the central nervous system processes signals originating from stressors to implement adaptive responses. This protective mechanism allows for the diversion of energetic resources from reproduction to an immune response [24]. Therefore, hypothalamic hypogonadism may potentially occur in the presence of any severe illness, including COVID-19, and cause temporary amenorrhea or infrequent menstruation. This might also explain why those who exhibit long-term symptoms of Ebola infection (post-Ebola syndrome, which is perhaps analogous to long COVID) have reported menstrual interruptions or irregularities [25].

Alternatively, or in addition, there may be more specific interactions between the reproductive system and SARS-CoV-2 infection. This may occur at the ovarian/endometrial level. Progesterone is predominantly an anti-inflammatory hormone [26] whose levels drop dramatically before menstruation, leading to an influx of inflammatory cells in the local endometrial environment and ultimately leading to the loss of the functional endometrium during menstruation [27]. Intense vasoconstriction of specialized endometrial spiral arterioles and activation of the local coagulation system act to limit menstrual blood loss. It is suggested that ACE2 receptors are present in ovarian and endometrial tissues [28] and, therefore, SARS-CoV-2 infection could hypothetically affect ovarian hormone production and/or the endometrial response during menstruation [29]. For example, altering the number/phenotype of endometrial leukocytes during or after SARS-CoV-2 infection could have the potential to affect menstrual blood loss. Previous research has shown that viral infection-induced immune activation was associated with the exacerbation of progesterone-related premenstrual symptoms [30]. In addition, COVID-19 infection has also been associated with endothelial cell dysfunction and alterations in the coagulation system, both critical components of endometrial function during menstruation, indicating a potential endometrial mechanism for menstrual disorders [27]. In light of these findings, it is important to consider other factors such as stress, lifestyle changes, lockdowns, restricted food availability, lack of personal medication for metabolic disorders, lack of sleep, and constant panic and stress during the pandemic. These factors might have played a more significant role in menstrual irregularities than the vaccine itself. For this reason, we compared the pre-pandemic and pre-vaccination phases (during which these factors were present) and found no significant differences in menstrual characteristics, thereby excluding the possibility that the observed alterations were correlated to other reasons related to the pandemic or to the SARS-CoV-2 infection itself, rather than the vaccination.

Treatments that have been recommended for disease symptoms include antipyretics and analgesics such as paracetamol, aspirin, and other non-steroidal anti-inflammatory drugs (NSAIDs). Additionally, corticosteroids and biotechnological drugs such as monoclonal antibodies have also been employed. There have been no reported studies in the literature on changes in menstrual cycle characteristics about COVID-19 therapies [31], although the mechanism of action of some of these interacts with the physiology of menstruation. NSAIDs affect the synthesis of endometrial prostaglandins and, through this mechanism, can reduce menstrual pain and blood loss [32,33]. Although some observational studies suggest that they may alter blood loss during menstrual flow, randomized controlled studies have refuted this hypothesis, concluding that they only reduce dysmenorrhea [33,34]. For hospitalized patients with COVID-19, one of the first effective treatments identified was dexamethasone, which can affect menstrual cycle characteristics and blood loss through cortisol actions [35]. The impact of certain treatments, including monoclonal antibodies, is unknown, although some studies on non-human primates have shown that anti-tumor necrosis factor (TNF) monoclonal antibodies may have some influence on the endometrium. Additionally, some treatments could negate cycle changes; for example, low oxygen saturation has been linked to low-grade inflammation and anovulatory cycles [36], but the potential impact of mechanical ventilation on cycles has not been explored.

This study presents several strengths in its investigation. One is the inclusion of a relatively large sample size, with 471 patients analyzed, which provided a substantial dataset for analysis, enhancing the statistical power of the study. We included women originating from various regions of Italy, avoiding the limitations of a single-center study that would have affected the generalizability of the results to a broader population. However, it is important to acknowledge some biases of the study, such as the retrospective design of the study and the lack of differentiation in the results based on the type of vaccine administered. Moreover, it is likely that the observed correlations between the COVID-19 pandemic and variations in menstrual cycles were more noticeable during the pandemic due to women more closely monitoring their health, menstrual cycle characteristics, and any minor changes, compared to the period before the pandemic. Although the reported changes in the menstrual cycle after vaccination are often temporary, the link between COVID-19 vaccination and menstrual cycle irregularities deserves to be investigated in further prospective studies.

## 5. Conclusions

Most women did not experience menstrual changes following COVID-19 vaccination. This offers reassuring data when informing women of reproductive age about the vaccine and potential menstrual irregularities. However, a small percentage did report cycle alterations, for which reason healthcare providers should consider informing women, through dedicated counseling, about the possible effects following vaccination. At the same time, it should be emphasized that consultation with a gynecologist is advised if disturbances last for several months or if different “warning” signs present themselves. Nevertheless, in most cases, a return to normality was observed after six months, with no clinically significant consequences. This is an important finding, especially for those women who wish to conceive and who might be discouraged by any changes to their cycle.

## Figures and Tables

**Table 1:** Comparison of the rhythm of the menstrual cycle.

		**Pre-pandemic rhythm**		Total
		regular	irregular	
**Rhythm during pre-vaccination pandemic phase**	regular	388 94.6%	8 13.1%	396 84.1%
	irregular	22 5.4%	53 86.9%	75 15.9%
Total		410 100.0%	61 100.0%	471 100.0%
		**Pre-pandemic rhythm**		Total
		regular	irregular	
**Rhythm after first vaccination**	regular	344 83.9%	16 26.2%	360 76.4%
	irregular	66 16.1%	45 73.8%	111 23.6%
Total		410 100.0%	61 100.0%	471 100.0%
		**Pre-pandemic rhythm**		Total
		regular	irregular	
**Rhythm after second vaccination**	regular	329 80.2%	18 29.5%	347 73.7%
	irregular	81 19.8%	43 70.5%	124 26.3%
Total		410 100.0%	61 100.0%	471 100.0%

**Table 2:** Comparison of the length of the menstrual flow.

		**Pre-pandemic length**			Total
		1–3 days	4–7 days	>7 days	
**Length during pre-vaccination pandemic phase**	1–3 days	44 95.7%	6 1.5%	0 0.0%	50 10.6%
	4–7 days	2 4.3%	399 98.0%	4 22.2%	405 86.0%
	>7 days	0 0.0%	2 0.5%	14 77.8%	16 3.4%
Total		46 100.0%	407 100.0%	18 100.0%	471 100.0%
		**Pre-pandemic length**			Total
		1–3 days	4–7 days	>7 days	
**Length after first vaccination**	1–3 days	35 76.1%	18 4.4%	0 0.0%	53 11.3%
	4–7 days	11 23.9%	380 93.4%	8 44.4%	399 84.7%
	>7 days	0 0.0%	9 2.2%	10 55.6%	19 4.0%
Total		46 100.0%	407 100.0%	18 100.0%	471 100.0%
		**Pre-pandemic length**			Total
		1–3 days	4–7 days	>7 days	
**Length after second vaccination**	1–3 days	33 71.7%	25 6.1%	0 0.0%	58 12.3%
	4–7 days	13 28.3%	366 89.9%	8 44.4%	387 82.2%
	>7 days	0 0.0%	16 3.9%	10 55.6%	26 5.5%
Total		46 100.0%	407 100.0%	18 100.0%	471 100.0%

**Table 3:** Comparison of the quantity of the menstrual flow.

		**Pre-pandemic quantity**			Total
		light	normal	heavy	
**Quantity during pre-vaccination pandemic phase**	light	37 82.2%	11 3.6%	3 2.4%	51 10.8%
	normal	5 11.1%	282 93.1%	18 14.6%	305 64.8%
	heavy	3 6.7%	10 3.3%	102 82.9%	115 24.4%
Total		45 100.0%	303 100.0%	123 100.0%	471 100.0%
		**Pre-pandemic quantity**			Total
		light	normal	heavy	
**Quantity during pre-vaccination pandemic phase**	light	41 91.1%	26 8.6%	19 15.4%	86 18.3%
	normal	1 2.2%	232 76.6%	20 16.3%	253 53.7%
	heavy	3 6.7%	45 14.9%	84 68.3%	132 28.0%
Total		45 100.0%	303 100.0%	123 100.0%	471 100.0%
		**Pre-pandemic quantity**			Total
		light	normal	heavy	
**Quantity during pre-vaccination pandemic phase**	light	37 82.2%	36 11.9%	22 17.9%	95 20.2%
	normal	5 11.1%	224 73.9%	17 13.8%	246 52.2%
	heavy	3 6.7%	43 14.2%	84 68.3%	130 27.6%
Total		45 100.0%	303 100.0%	123 100.0%	471 100.0%

**Table 4:** Comparison of the menstrual cycle rhythm and menstrual flow duration and quantity between the pre-pandemic and the pre-vaccination phases.

	**Pre-pandemic cycle rhythm and** **pre-vaccination pandemic rhythm**	
Sample size	471	
Exact two-tailed significance	0.108 ^a^	
McNemar’s test was used. ^a^ Binomial distribution.		
	**Pre-pandemic flow duration** **Pre-vaccination pandemic duration**	**Pre-pandemic flow quantity** **Pre-vaccination pandemic quantity**
Z	−1.604	−1.665
Asymptotic two-tailed significance	0.119 ^a^	0.096 ^a^
The Wilcoxon test was used. ^a^ Based on positive ranks.		

**Table 5:** Percentage of transition from regular to irregular cycle (out of a total of 471 patients examined) in the pre-pandemic phase compared to the other phases.

Variable	Phase	Percentage of Transition from Regularity to Irregularity	Comparison and *p*-Value
Rhythm	A. During pre-vaccination pandemic phase	5.4%	A vs. B *p* < 0.001
	B. After first vaccination	16.1%	A vs. C *p* < 0.001
	C. After second vaccination	19.8%	B vs. C *p* = 0.139
Length		From 4–7 days to 1–3 days	
	A. During pre-vaccination pandemic phase	1.5%	A vs. B *p* = 0.008
	B. After first vaccination	4.4%	A vs. C *p* < 0.001
	C. After second vaccination	6.1%	B vs. C *p* = 0.242
		From 4–7 days to >7 days	
	A. During pre-vaccination pandemic phase	0.5%	A vs. B *p* < 0.024
	B. After first vaccination	2.2%	A vs. C *p* < 0.001
	C. After second vaccination	3.9%	B vs. C *p* = 0.129
Quantity		From normal to light	
	A. During pre-vaccination pandemic phase	3.6%	A vs. B *p* = 0.001
	B. After first vaccination	8.6%	A vs. C *p* < 0.001
	C. After second vaccination	11.9%	B vs. C *p* = 0.095
		From normal to heavy	
	A. During pre-vaccination pandemic phase	3.3%	A vs. B *p* < 0.001
	B. After first vaccination	14.9%	A vs. C *p* < 0.001
	C. After second vaccination	14.2%	B vs. C *p* = 0.761

Alpha level adjusted for multiplicity (Bonferroni correction) = 0.017.

**Table 6:** Phase of the menstrual cycle during which patients with irregular rhythm—after the first vaccination—received their first vaccination.

Menstrual Cycle Phase	Patients (*n*)	Patients (%)
Early proliferative phase	17	15.3
Advanced proliferative phase	65	58.6
Secretive phase	29	26.1
Total	111	100.0

**Table 7:** Phase of the menstrual cycle during which patients with irregular rhythm—after the second vaccination—received their second vaccination.

Menstrual Cycle Phase	Patients (*n*)	Patients (%)
Early proliferative phase	26	21.0
Advanced proliferative phase	68	54.8
Secretive phase	30	24.2
Total	124	100.0

## Data Availability

Data are contained within the article.

## Supplementary Materials

The following supporting information can be downloaded at: https://www.mdpi.com/article/10.3390/jcm12247699/s1. File S1: Menstrual Cycle Changes and COVID-19 Vaccination: Possible Link or Overestimated?
