# Supplementary material for: Effects of SARS-CoV-2 Vaccination on Menstrual Cycle: An Italian Survey-Based Study
Source: J Clin Med. 2023 Dec 15;12(24):7699. doi: 10.3390/jcm12247699 (PMC10744112; doi:10.3390/jcm12247699)
Supplement: Supplementary file 1 [file jcm-12-07699-s001.zip › jcm-2708687-supplementary.pdf]

Menstrual Cycle Changes and COVID-19 Vaccination: Possible Link or Overestimated?

Vaccination against SARS-CoV-2 has played a critical role in controlling the spread of the pandemic. The main side effects of SARS-CoV-2 vaccination include fever and fatigue, but also potential impacts on menstrual cycles are to be determined. Given limited studies suggesting menstrual changes post-vaccination, this survey investigates the correlation between COVID-19 vaccines and menstrual cycle changes.

The questionnaire is completely anonymous, and its completion will take about 3 minutes of your time.

You will be asked questions about your menstrual cycle and any COVID-19 vaccinations through a survey.

The project does not involve the transfer of personal information. By agreeing to complete the questionnaire, you give your consent to the analysis of the data in a completely anonymous form. The data will converge into a database and will be used only to transmit project results and research. The database will be available to the research team only for the time necessary for processing, interpreting the data, and drafting publications. The processing of personal data is managed in accordance with Article 13 of the General European Regulation No. 679/2016, respecting privacy and anonymity.

Participation in the research is voluntary and free of charge.

Only one answer per question can be indicated.

1. Indicate your age.
    - __ years
2. Have you received both doses of the SARS-CoV-2 vaccine?
    - Yes
    - No
3. What was the first dose of the SARS-CoV-2 vaccine you received?
    - Pfizer-BioNTech
    - Moderna
    - AstraZeneca
    - Johnson & Johnson
    - Other
4. What was the second dose of the SARS-CoV-2 vaccine you received?
    - Pfizer-BioNTech
    - Moderna
    - AstraZeneca
    - Johnson & Johnson
    - Other
5. Are you undergoing any form of hormonal treatment?
    - Yes
    - No
6. Define the rhythm of your menstrual cycle before the pandemic:
    - Regular
    - Irregular
7. Are you pregnant?
    - Yes
    - No
8. Have you ever contracted SARS-CoV-2?
    - Yes
    - No
9. In which Italian region do you live?

- Abruzzo
- Aosta Valley
- Apulia
- Basilicata
- Calabria
- Campania
- Emilia-Romagna
- Friuli-Venezia Giulia
- Lazio
- Liguria
- Lombardy
- Marche
- Molise
- Piedmont
- Sardinia
- Sicily
- Tuscany
- Trentino-Alto Adige/Südtirol
- Umbria
- Veneto

10. What is your highest level of education?
    - University degree
    - High school diploma
    - Completed middle school
    - Did not complete middle school
11. During the pandemic, your work and/or study activity:
    - Remained unchanged
    - Decreased
    - Increased
12. During the pandemic, your monthly earnings:
    - Remained unchanged
    - Decreased
    - Increased
    - I lost my job due to the pandemic
    - I do not receive a salary
13. During the pandemic, your body weight:
    - Remained unchanged
    - Decreased
    - Increased
14. During the pandemic, your physical activity:
    - Remained unchanged
    - Decreased
    - Increased
15. During the pandemic, your stress level:
    - Remained unchanged
    - Decreased
    - Increased
16. Have you had pregnancies?
    - Yes, resulting in both live births and abortions
    - Yes, only resulting in live births
    - Yes, only abortions
    - No

17. How would you define the rhythm of your menstrual cycle during the pandemic before vaccination?
    - Regular (every 25-35 days)
    - Irregular
18. How would you define the rhythm of your menstrual cycle after the first vaccination?
    - Regular (every 25-35 days)
    - Irregular
19. How would you define the rhythm of your menstrual cycle after the second vaccination?
    - Regular (every 25-35 days)
    - Irregular
20. Indicate the duration of your menstrual cycle during the pandemic before vaccination:
    - 1-3 days
    - 4-7 days
    - > 7 days
21. Indicate the duration of your menstrual cycle after the first vaccination:
    - 1-3 days
    - 4-7 days
    - > 7 days
22. Indicate the duration of your menstrual cycle after the second vaccination:
    - 1-3 days
    - 4-7 days
    - > 7 days
23. Indicate the amount of your menstrual cycle during the pandemic before vaccination:
    - Normal
    - Light
    - Heavy
24. Indicate the amount of your menstrual cycle after the first vaccination:
    - Normal
    - Light
    - Heavy
25. Indicate the amount of your menstrual cycle after the second vaccination:
    - Normal
    - Light
    - Heavy
26. If you reported an irregular menstrual cycle rhythm after the first vaccination, in which phase of the menstrual cycle were you when you received the first dose of the vaccine?
    - Between 1st and 3rd day (early proliferative phase of the menstrual cycle)
    - Between 4th and 14th day (late proliferative phase of the menstrual cycle)
    - After the 14th day (secretory phase of the menstrual cycle)
27. If you reported an irregular menstrual cycle rhythm after the second vaccination, in which phase of the menstrual cycle were you when you received the second dose of the vaccine?
    - Between 1st and 3rd day (early proliferative phase of the menstrual cycle)
    - Between 4th and 14th day (late proliferative phase of the menstrual cycle)
    - After the 14th day (secretory phase of the menstrual cycle)
